# Soil health pilot study in England: Outcomes from an on-farm earthworm survey

**DOI:** 10.1371/journal.pone.0203909

**Published:** 2019-02-20

**Authors:** Jacqueline L. Stroud

**Affiliations:** Sustainable Agricultural Systems Department, Rothamsted Research, Harpenden, United Kingdom; University of Roehampton, UNITED KINGDOM

## Abstract

Earthworms are primary candidates for national soil health monitoring as they are ecosystem engineers that benefit both food production and ecosystem services associated with soil security. Supporting farmers to monitor soil health could help to achieve the policy aspiration of sustainable soils by 2030 in England; however, little is known about how to overcome participation barriers, appropriate methodologies (practical, cost-effective, usefulness) or training needs. This paper presents the results from a pilot #60minworms study which mobilised farmers to assess over >1300 ha farmland soils in spring 2018. The results interpretation framework is based on the presence of earthworms from each of the three ecological groups at each observation (20 x 20 cm x 20 cm pit) and spatially across a field (10 soil pits). Results showed that most fields have basic earthworm presence and abundance, but 42% fields may be over-worked as indicated by absence/rarity of epigeic and/or anecic earthworms. Tillage had a negative impact (*p <* 0.05) on earthworm populations and organic matter management did not mitigate tillage impacts. In terms of farmer participation, Twitter and Farmers Weekly magazine were highly effective channels for recruitment. Direct feedback from participants included excellent scores in trust, value and satisfaction of the protocol (e.g. 100% would do the test again) and 57% would use their worm survey results to change their soil management practices. A key training need in terms of earthworm identification skills was reported. The trade-off between data quality, participation rates and fieldwork costs suggests there is potential to streamline the protocol further to #30minworms (5 pits), incurring farmer fieldwork costs of approximately £1.48 ha^-1^. At national scales, £14 million pounds across 4.7 M ha^-1^ in fieldwork costs per survey could be saved by farmer participation.

## Introduction

There is now a significant interest in sustainable soil management and policy in England to achieve the Department of Farming and Rural Affairs (DEFRA) aspiration of sustainable soils by 2030. A sustainable arable agricultural system is considered to have both sustainable crop production for food security and a ‘healthy’ soil for soil security. However, there have been few soil surveys to inform both land managers and policy makers about the state of farmland soil health in England to best support evidence-based decision making.

Over the past decade there have been a number of successful public soil surveys in England using earthworm populations including the Open Air Laboratories Soil and Earthworm Survey which included 0.4% sites in arable fields[1]; the Natural England earthworm surveys which included 1.8% sites in arable fields[2]; and a school citizen science invertebrate survey (0% sites in arable fields)[3]. Although earthworms are a primary candidate (out of 183 potential biological indicators) for national soil health monitoring[4], there has been limited farmer participation to date. Mobilising farmers to monitor soil health could be an effective way to improve the national sustainability of soil management. For example, the ‘monitoring effect’ where farms taking part in monitoring activities improve their biodiversity faster than farms not taking part in monitoring[5], fits well with sustainable soil policy aspirations for UK agriculture.

Arable soils typically contain 150–350 earthworms per m^2^ and high populations (>400 earthworms per m^2^) are linked to significant benefits in plant productivity, including cash crops such as wheat [6]. There are three ecological functional groups: epigeic earthworms break down surface crop residues and their presence is linked to the breeding season success rates of the song thrush (*Turdus philomelos*), the latter whose populations have rapidly declined in England[7]. Anecic earthworms incorporate surface organic matter into the soil; and support water drainage for plant production[8] and deep crop rooting[9]. UK endogeic earthworm species mix organic and mineral components together to form stable aggregates which benefit spring crop emergence and carbon sequestration[10]. In this way, earthworms support both food production, but also wider ecosystem services associated with soil security. There is no evidence that earthworm biodiversity is constrained in the UK[11], and invasive flatworms which are earthworm predators are largely geographically restricted to Western Scotland and Ireland[12]. Thus, arable soil management is a key factor controlling the relative abundance of these ecological functional groups.

In terms of arable soil management, both epigeic and anecic earthworm species are highly vulnerable to conventional tillage[13], meaning earthworm community structures could be used to indicate over-worked soils. Crop establishment practices have been dominated by intensive mechanical cultivation for decades[14], and this continues to be the principal soil management practice for establishing arable crops in England [15]. It is well known that tillage has an adverse effect on the environmental services provided by soils [16]. Over-cultivation impacts soil biological, physical and chemical properties, for example, causing a decline in surface-feeding earthworms to local extinction levels[13, 17], reduces water stable aggregation which increases the risk of erosion and nutrient losses, and may decrease soil organic carbon levels with implications for climate change[18]. It is unclear as to the extent organic matter management can mitigate the effects of tillage, as the impact of these management activities is subject to local conditions[17].

To date, the use of earthworms in national monitoring schemes has been held back by the absence of a standardised methodology [4]. For example, all three ecological earthworm surveys in England over the past decade have used a different methodology [1–3]. These methods differ from the ISO 23611–1 earthworm assessment method which includes formalin as a vermifuge, precluding its application in citizen science projects. A limitation of the largest international survey of farmland earthworm populations (EU FP7 BioBio) was the skilled labour based protocol and high labour cost (on average 4.8 person days (£3 k) per farm for earthworm fieldwork alone, not including taxonomic identification)[5].

The ultimate aim of monitoring is to cost-effectively convey robust information to those who are expected to use it [19]; essentially the trade-off between data quality, practicability, cost and usefulness. The principal cost of monitoring is labour; for which the UK has the highest person day costs in the EU [5]. Research from the EU FP7 BioBio project indicated significant cost reductions (46%) could be achieved if farmers could be mobilised to assess their own farms; however, key research areas include how to overcome participation barriers; the development of protocols that require lower technical expertise; identification of training needs and quantifying sampling bias [5]. To date, one small study assessed the usefulness of ‘earthworms’ (numbers and species) for farmland biodiversity assessments to administrators, farmers and consumer groups, with earthworms ranked 5^th^ (out of 6 parameters) by all groups [19].

The aim of the #60minworms pilot study was to support farmers to monitor their own field(s) and generate results that are useful to their soil management decisions–specifically to help identify potentially over-worked soils. The objective of this research was to address the gaps in on-farm earthworm monitoring are provide the first insights into the soil biology of farmland soils in England.

## Methods

### Farmer recruitment and engagement

**Fig 1:**
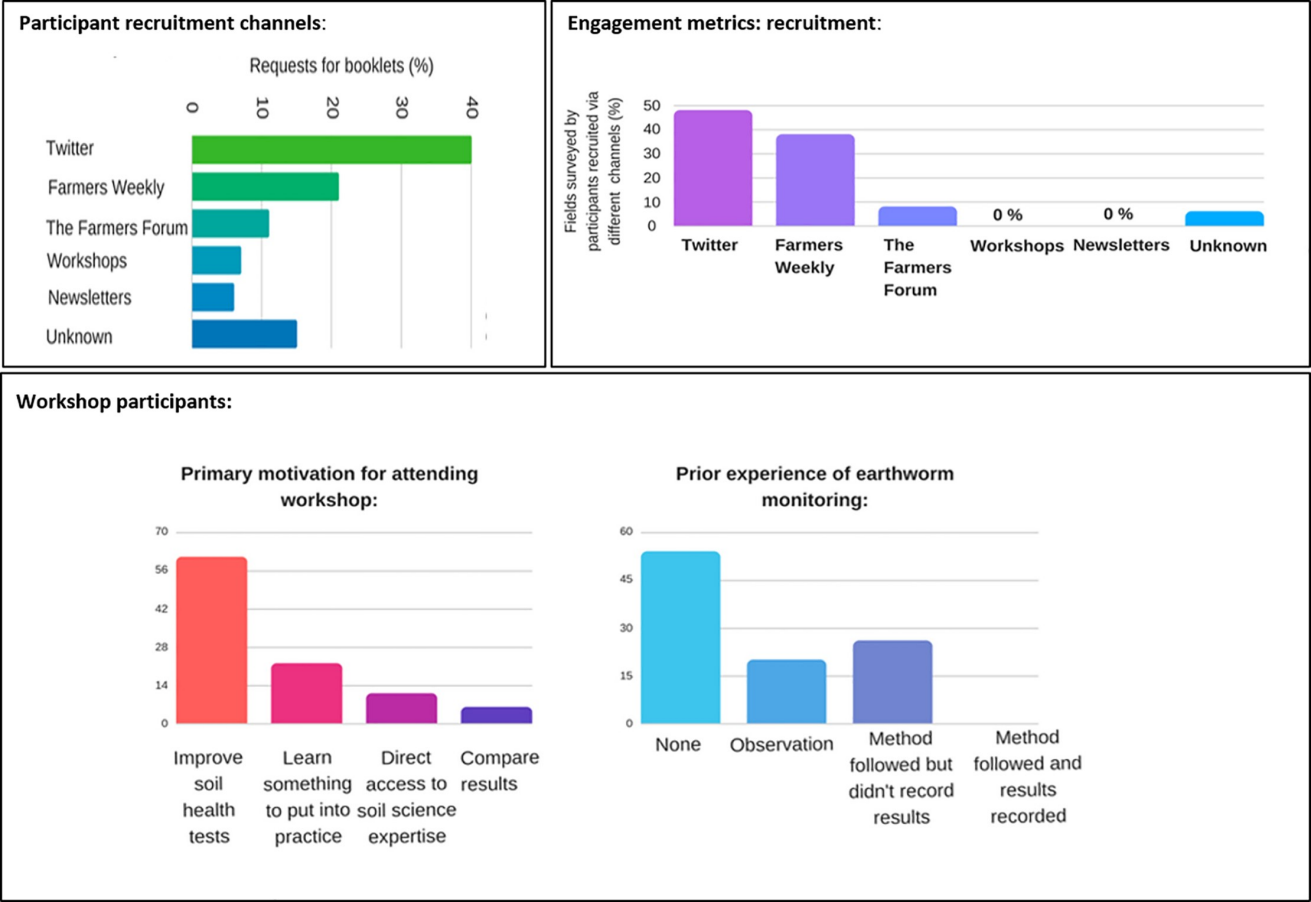
Recruitment, participation and engagement in the #60minworms survey. The key mobilisation routes were through Twitter and Farmers Weekly, the survey attracted participants with no earthworm monitoring experience and the primary feedback preference was a workshop.

The #60minworms pilot study (100 fields target) ran between the 15^th^ March– 30^th^ April 2018 to provide a 6-week window for Spring earthworm sampling (Fig 1). There was no need for ethical approval as this was undertaken by volunteers (farmers) on their privately-owned land (farms). There was no data collected that could lead to the identification of the participants e.g. location, or any information collected about the participants (e.g. gender, age), and the data sheet was either posted or a photograph was emailed by the volunteer to the scientist so there was no metadata associated with these results. The participants provided written consent to receive the results via email, and farms visited by the scientist with interesting earthworm findings was by invitation only. The age of the volunteers was not collected but based on social media posts by the volunteers using the #60minworms hashtag, both adults and families with young children participated.

Stroud designed the survey booklets (S1 Protocol), printed 300 copies and numbered each one in order to quantify recruitment and participation levels through different channels (Fig 1). Channels for recruitment included direct promotion of #60minworms at two soil health workshops, one in Southern England (Catchment Sensitive Farming event, Hertfordshire) and one in Northern England (Agronomy company event, Northumberland) with a combined audience size of 114 people. Indirect recruitment included Twitter, with the initial tweet via @rothamsted (host institute, >10k followers). Stroud started a thread on Farmers Forum (>32, 000 members) to recruit volunteers; and the worm survey was featured in national farmer press (Farmers Weekly 16^th^ March 2018, circulation >59, 000). The survey was mentioned in newsletters by stakeholders (unknown circulation). Some requests were made to general Rothamsted staff (via phone or email) and the channel was not recorded. There were 227 booklet requests and these were only distributed as a printed copy, either directly (at workshops) or following a request (via telephone, email or Twitter) and posted to potential participants. Participant recruitment and engagement was encouraged via social media posts over the 6-weeks using Twitter @wormscience (scientist, 1.7k followers) and @Soil_Security (project funder, 1k followers). Whilst the response was generally positive, negative responses on Twitter included farmers reporting taking part in scientific surveys but never receiving feedback. These people identified that a workshop was their primary feedback preference. All the participants (i.e. those that sent in results) were invited by email to take part in the #60minworms workshop on the 3^rd^ May 2018 held at Rothamsted. Particpants were asked if there were any specific questions/activities they would like to be covered at the workshop and an earthworm masterclass (species level earthworm identification) and visit to Broadbalk was requested and facilitated.

### Pilot method for field scale earthworm sampling

The #60minworms method was designed around the presence of earthworms in the field, enabling a rapid ‘traffic-light’ based interpretation. The participants required five pieces of equipment to perform the survey: a garden fork or spade (depending on the soil type) to dig the soil pit, a ruler (as 20 cm x 20 cm x 20 cm size pits were needed), a mat (to put the soil on for hand-sorting *in-situ*), a pot with a lid (to stop earthworms escaping) plus a small volume of water (so the earthworms do not dry out) and the results booklet (including a simple earthworm key) with a pen. A timer was recommended to complete the hand-sorting within 5 minutes, unless the soil was too wet or compacted to sort efficiently and time was increased to 10 minutes. Thus, the equipment and consumable costs were negligible; and, an experienced sampler could generally complete the survey in 60 minutes. The procedure is to dig a 20 cm x 20 cm x 20 soil pit and place the soil on the mat. The soil is hand-sorted, placing each earthworm into the pot. Once the soil has been sorted, the total number of earthworms (both adults and juveniles) were counted and recorded. The earthworms were separated into adults (for further analysis) and juveniles (returned to the pit). Adult earthworms were separated into an ecological functional group (epigeic, endogeic or anecic) using a simple key. The total numbers of epigeic (small red worms), endogeic (pale or green worms) or anecic (heavily pigmented, large worms) adults were recorded for each pit. There are high levels of cryptic diversity within UK earthworm species[20], thus species level assessments are beyond the scope of this agricultural soil health assessment. After analysis, the adult earthworms were returned to the pit. This was repeated 10 times via a W-style sampling pattern across the cropped field. The data was recorded in the results booklet (S1 Protocol), and these were either posted or photographed and emailed back for analysis.

### Quality control

To address some of the common concerns relating to earthworm analyses, the seasonal reproducibility was tested on nine AHDB strategic farm fields (eight arable and one grass field) in October 2017 and April 2018. To assess the reliability of 10 or fewer soil pits per field; 20 soil pits per field (n = 9 fields) were measured. To assess the accuracy of hand-sorting earthworms in 5 minutes, sorted soil was re-sorted for 5 minutes and earthworms were collected for further analyses. This was performed by three volunteers on nine fields (range of soil textures and crop types) (n = 27 pit resorted) in April 2018. To indicate year-on-year variability, previous scientific field trial based earthworm surveys[21] (using the identical soil pit size and hand sorting methods), with at least two years of data were re-analysed (to remove vermifuge data and categorise the species into their ecological groupings), and recalculated on a per pit basis using the likelihood formula.

The 10 participants with either the most depleted or exceptional earthworm results were contacted to arrange a field/farm visit to validate the result, obtain soil texture information and receive informal verbal feedback on the method and usefulness of the results. A total of 10 fields were visited in the South West, South East, East and Midlands.

### Data analysis

The results interpretation framework is based on the earthworm presence and abundance for each observation (20 x 20 cm x 20 cm pit) across a field (10 soil pits). There were five categories to quantify earthworm presence and abundance: (a) ‘widespread’–how many soil pits were earthworms (juveniles or adults) found, b) epigeic, (c) endogeic, (d) anecic–how many soil pits earthworms from each of the ecological groups (adults only) were found, and (e) how many soil pits where high numbers of earthworms (≥16 worms) were found. The results can be calculated via a simple formula:

$$Earthworm\;community=(\frac{a,b,c,d\;or\;e}{Total\;number\;of\;soil\;pits})\times 100$$

Where:

(a) Total number of soil pits with ≥1 earthworm (juveniles or any adults below),
(b) Total number of soil pits with ≥1 adult epigeic earthworm,
(c) Total number of soil pits with ≥1 adult endogeic earthworm,
(d) Total number of soil pits with ≥1 adult anecic earthworm,
(e) Total number of soil pits with high numbers (≥ 16 earthworms per pit, ≥400 earthworms per m^2^) of earthworms (total number including all juveniles and adults).

The traffic light system interpretation used was a red ‘unlikely’ category (<33%), the amber, ‘possibly’ category (>33–66%) and the green ‘likely’ category (> 66%), and is reported on a field basis (Table 1).

**Table 1:** The interpretation framework is based on the presence of earthworms for each observation (one soil pit) across a field (10 soil pits).

% Occurrence	Interpretation:	Traffic light colour	Threshold
0–1	Exceptionally unlikely	Red	Concern
1–10	Very unlikely	Red	Concern
10–33	Unlikely	Red	Sub-optimal
33–66	Possibly	Amber	Satisfactory
66–90	Likely	Green	Good
90–99	Very likely	Green	Good
99–100	Almost certain	Green	Good

The threshold of concern for each category was based on ≤1/10 soil pits (≤10%) containing at least one earthworm (a), an earthworm from each ecological group (b-d) and high numbers (e) as this provides little evidence of earthworm presence and abundance. In comparison, the satisfactory/good threshold means there is evidence for earthworm presence and abundance: *category a*—earthworms are widespread across the field to support plant productivity and ecosystem services. *Categories b–d*–earthworms present have capabilities down the soil profile, and as an adults’ lifespan is in the order of years, and given their reproduction capacity, there is evidence for previous duration and future sustained capability). *Category e*–earthworm abundance at these high levels is associated with a significant impact on plant productivity.

Earthworm numbers were not of primary interest in this survey because the interpretation is dependent on fertiliser usage, soil type, crop type etc[6], but to calculate the average number of earthworms per hectare the following formula was used:

(f) *Earthworms per hectare* = (*mean number of earthworms per pit* × 25) × 10000

Whilst the results (simple percentages) could be calculated by the participants, they were requested to either post or email a copy of their findings, and include basic field management details including field name, size, crop, tillage (notill, minimum tillage and ploughed), and Yes/No answers to organic matter management: residue retained, cover cropping and whether an organic waste e.g. compost had been used this year, in order to inform on general soil management practices and earthworm results.

Following the submission of all the data, Genstat (18.2.0.18409, 18^th^ addition, VSN International Ltd., UK) was used to perform one-way ANOVAs to assess trends in earthworm populations and soil management practices. Labour cost estimates were calculated using a £:€ exchange rate of 1.12; in order to translate private agency skilled worker (€89.75 h^-1^) and farmer (€28.39 h^-1^) [5]. To calculate costs at farm, regional and national scales, DEFRA official statistics (February 2018) were used [22]. The survey data was compared against the earthworm soil health thresholds proposed in this paper.

### #60minworms workshop

All the participants received a report on their earthworm populations by email and were invited to take part in the #60minworms workshop on the 3^rd^ May 2018 at Rothamsted. The workshop was based around a ClikaPad audience response system to enable an anonymous, real-time vote to 30 questions to quantify sampling design bias, method compliance, competence, usefulness, satisfaction and future developments; followed by an open discussion of each answer. After this classroom based activity, a practical earthworm identification master class was held at the farm adjacent to the Broadbalk field (at participants request) which involved identifying earthworms to species level using the OPAL key[23]. The outcomes from this workshop were adopted to make the new Agricultural and Horticultural Development Board (AHDB) factsheets ‘How to count worms’ freely available as printable leaflets in June 2018, with an initial print run of 2000 copies, distributed at agricultural events such as Cereals (leading technical event for the arable industry with up to 20,000 visitors) and AHDB strategic and monitor farm events (24 sites around the UK) (S1 Protocol).

## Results

### Recruitment and engagement of farmers

The inial recruitment tweet via @rothamsted (host institute, >10k followers) received 28, 401 impressions (number of Twitter accounts where the tweet was seen). The Rothamsted #60minworms project page had 733 views, with an average page time of 3 minutes and 22 seconds. The Farmers Forum post recieved 26 responses and 1134 views, with futher discussion and reviews of the method posted by participants. The @wormscience account had a total of 171, 600 impressions over this period, with a maximum of 23, 793 per post and engagement rate of 8.9%. Approximately 40% Twitter recruits used ‘#60minworms’ to post photos of fieldwork and reviews of the method on Twitter. On-going communication with participants, for example responding to sampling and method support (earthworm identification) requests, was principally via Twitter and email (an average of 15 interactions per day over the 42-day sampling window), and led to the creation of additional online resources such as a YouTube #60minworms demonstration (218 views, 305 minutes wated in total with an average view time of 1 minute and 40 seconds). This also led to the change in ‘traffic-light’ shading in results to improve accessibility to colour blind participants. The top three questions were (1) when to sample (soil temperature); (2) suitable for children to participate? and (3) sample specific crop/soil type required?

A total of 126 fields were surveyed, which was 1318 ha of farmland soils. Engagement rates ranged from 0% (workshops, newsletters) to 55% (Twitter) and 40% (Farmers Weekly). Interestingly the Farmers Weekly participants sent in multiple field results (the maximum number of surveys that were returned by one participant was seven).

A total of 11% #60minworms participants attended the workshop (Fig 1). The majority of workshop participants (56%) ranked their knowledge of earthworms as ‘below average’ and the principal reason for workshop attendance was to ‘improve soil health assessments’ (61%), with minor interests in ‘learn something to put into practice’ (22%), ‘direct access to soil science expertise’ (11%) and ‘comparing results to others’ (6%). The workshop participants represented a full diversity of soil management practices, including the highest ranked field and the lowest ranked fields in the survey, organic and conventional management, participants across the full spectrum of tillage (zerotillage, mintill and plough-based). In terms of prior earthworm survey experience, the majority (54%) had never done anything like this before, where 20% had noticed a difference in worms (nothing formal) and 26% had followed a method but didn’t record the results (semi-formal). No (0%) participants had taken part in formal monitoring (following a method and recording results). In terms of their results, 19% were pleasantly surprised, 25% results were as expected, 13% were worse than expected and 50% participants didn’t know what to expect. These findings indicate that there was no significant bias in the workshop participants in either soil management or results interpretations.

### Cost and usefulness of the #60minworms survey

Qualitative feedback was provided directly (email, twitter posts or verbal) with ‘added value’ of the worm survey including the detection of compaction problems, anaerobic/slowly degrading organic materials, linear decline in earthworms across a field leading to soil chemistry assessments, predator problems (moles) and one participant, who did not complete the survey due to being alarmed by initial findings sought assistance from a commercial soil health app (sectormentor). Negative feedback included the ‘traffic-light’ being difficult to interpret and the 60 minute duration feedback was mixed, some people commented it was achievable, others highlighted the intial few pits took the longest until they had ‘got their eye in’, where others with >20 worms per soil pit stated the 60-minutes duration was unachievable.

Quantifiable feedback was provided by the workshop participants. Most participants (77%) reported spending 5-mins hand-sorting each soil pit, enabling completion within 60 minutes. The number of samples was fixed at 10 replicates, but field surveys ranged between 2 to 80 hectares (average observation was 1.08 ± 0.08 pit per hectare) and the longest reported survey took 3 hours. Using the person (farmer) day costs in the UK[5], where the majority (66%) of participants performed the #60minworms analysis alone means the typical farm labour costs were €28 (£25). A total of 34% participants completed the survey with fieldwork support provided by up to 3 people, increasing the cost to €84 (£75) per field. The real farm labour costs (in-kind) for the 126 field #60minworm pilot field study can therefore be estimated to be in the order of €5928 (£5300); which on a per hectare basis is €4.50 (£4).

**Fig 2:**
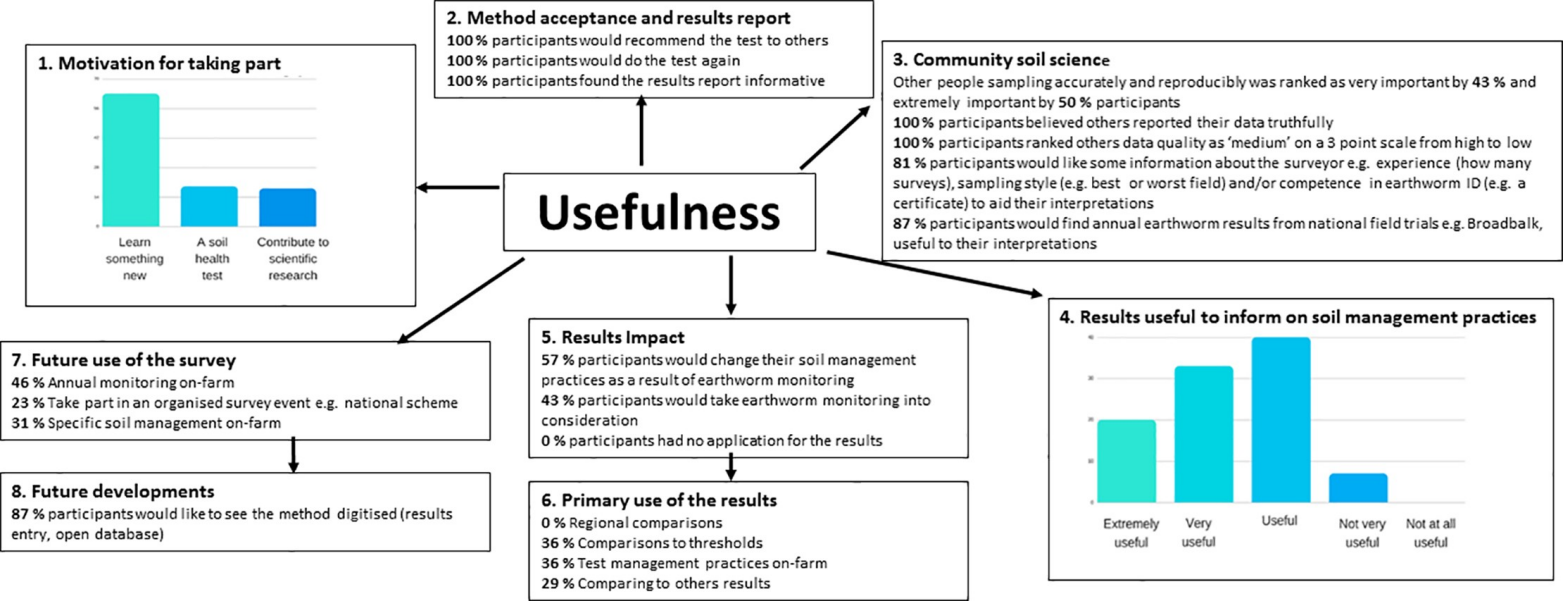
Usefulness of the #60minworms survey to farmers. Feedback included trust, value and satisfaction in the protocol by participants (100% would do the test again) and an extremely high interest (>85%) in community science (including other participants and scientists) with a key use in comparing results.

There were a range of motivations for taking part in the #60minworms survey, and excellent scores in value, trust and satisfaction of the method (Fig 2); for example, 100% of the participants would do the #60minworms survey again. There were very high scores for community science in every category; where 100% participants would recommend the survey to others, 93% participants rated other participants’ competence was very important and 87% participants would use of scientific field trials to aid their interpretations; which corroborated with the high (29%) primary use of results would be to compare their results to others (Fig 2). Further, most participants would use the survey to compare soil management practices on-farm (36%). This results was in agreement with the finding that #60minworms participants often performed multiple field surveys (up to seven fields) and would change their soil management practices based as a result earthworm monitoring results (57% participants) (Fig 2). There was no interest in regional trends in earthworm populations, only on-farm, between farm, national scientific field trials and threshold values to aid interpretation (Fig 2).

### Quality control and application

**Fig 3:**
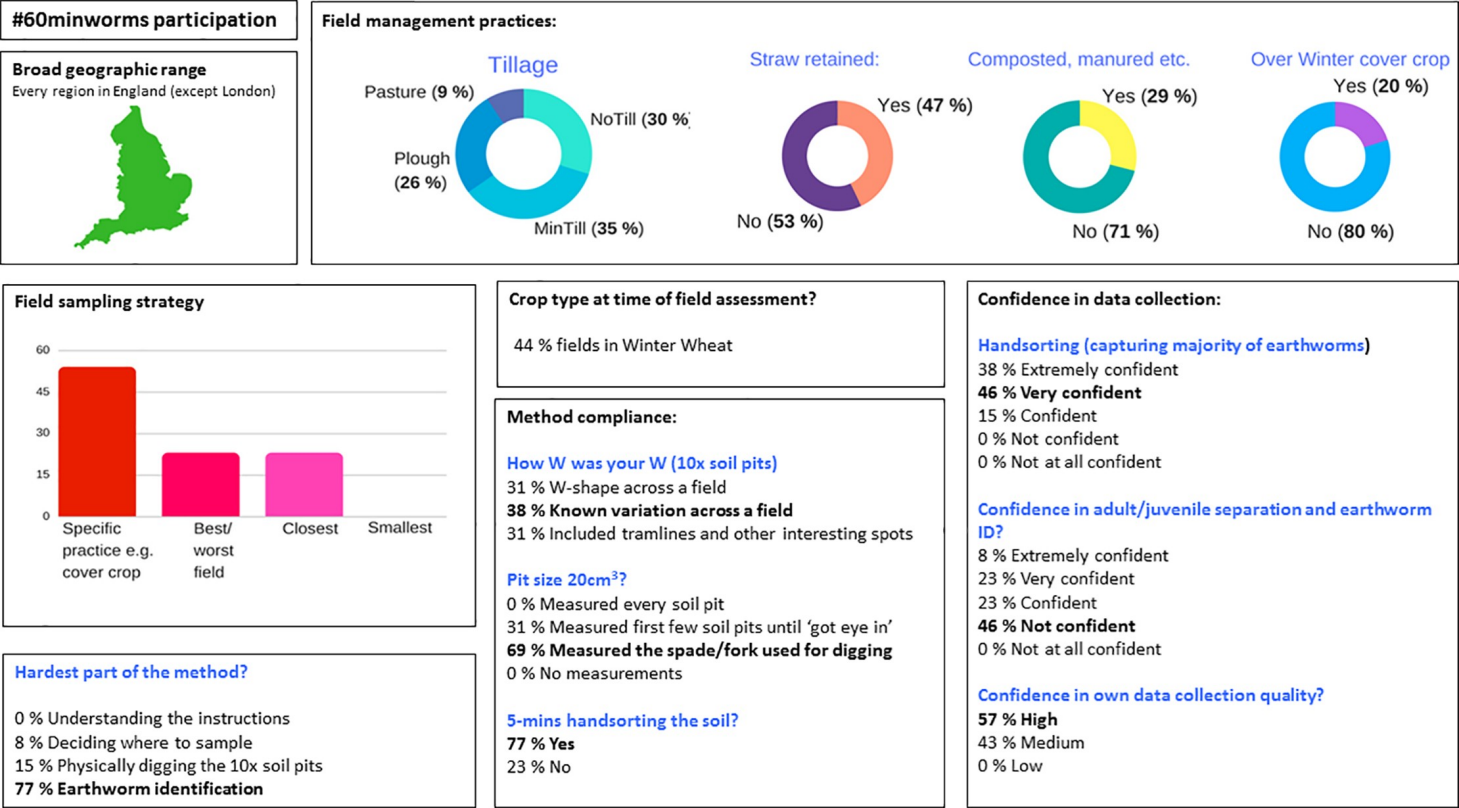
#60minworms survey participation. There was a broad geographic spread over England and a range of field management practices. There was little indication of bias in sampling strategy, problems in compliance or results quality, but there was a key training need in terms of earthworm identification skills.

There was full geographic coverage in England and a range of management practices surveyed (Fig 3). Choosing the smallest field was not a sampling strategy by any participant, and good levels of compliance were recorded, for example, all participants measured the size of their soil pit(s). A key training need in earthworm identification skills was identified (Fig 3). Farmers reported a problem capturing deep burrowing *Lumbricus terrestris* anecic earthworms which could be solved by amending the method to include a tick box for the presence of middens/characteristic large vertical burrows. There are three common anecic earthworm species in England (*L*.*terrestris*, *A*.*longa* and *A*. *nocturna*), and middens are a good indicator of *L*.*terrestris*[24–30], the earthworm most sensitive to conventional tillage [13].

The intensive sampling at the AHDB strategic farm fields also measured the accuracy of 5-minute soil pit handsorting for earthworms. Resorting soil for a further 5-minutes led to an additional 1.6 ± 0.17 earthworms per pit per field (regardless of earthworm population size), ranging in biomass from 0.05–0.429 g per earthworm, of which 91% were juveniles; meaning the underestimation of 40 worms per m^2^ (or 400, 000 ha^-1^) on each field. The variability of earthworm populations over annual scales was high for earthworm numbers (S1 Table); but the presence (or absence) of each ecological group was consistent (S1 and S2 Tables). Comparing results at 20, 10 and 5 sampling pits per field; 10 sampling pits would incur an error of 16% in categorizing the earthworm groups; of which 4% would be a false negative (i.e. 0%, no sightings on that ecological group which is uncommon rather than absent); five sampling pits per field would incur an error of 33% in categorizing the earthworm groups, of which 15% would be a false negative.

### #60minworms survey results

Earthworm counts within a 10-pit field survey ranged by 6.4-fold, from a minimum 1.3 to a maximum difference of 28-fold. The average earthworm field population (total number of earthworms including adults and juveniles) was 2.4 ± 0.4 million worms ha^-1^ (approximately 9 worms per soil pit) and ranged by 100-fold, between 0.75 to 7.3 million worms ha^-1^. The field characteristics of the top and lowest 10 populations of earthworms shared soil textures, tillage and field management practices (S3 Table). Tillage significantly (*p <* 0.05) impacted the general earthworm presence, epigeic presence, anecic presence, presence of hotspots and number of earthworms per hectare (S1 Fig, S4 Table). Organic matter management included straw retention, cover cropping or manuring (including animal manures, compost, anaerobic digestate, humic substances or biosolids). The only significant impact on the numbers of earthworms was straw retention (*p =* 0.04), S4 Table. Cover cropping, significantly impacted the presence of anecic earthworms (*p =* 0.03), (S2 Fig, S4 Table).

A total of 77% fields had a 100% presence of earthworms (at least 1 earthworm per pit), with the lowest presence recorded at 30% for one field. There were no sightings of epigeic earthworm on 21% fields, and anecic earthworms on 16% fields (S5 Table), with a further 8–11% fields have rare sightings of these groups (10% presence). There was a good (≥ 67% presence) of endogeic earthworms on most fields (S5 Table); and a good presence of all three ecological groups together on 15% fields. Earthworm hotspots (≥16 earthworms per pit) were uncommon; 46% fields had no earthworm hotspots, where a good presence of hotspots was detected on 13% fields. Overall, 42% fields had sub-optimal earthworm populations, defined as ≤10% presence for at least one ecological group, providing little evidence for the spatial and temporal presence of epigeic, endogeic and/or anecic earthworms.

### Trade-offs between data quality, participation rates and cost

The aim of #60minworms was to indicate soils at risk of being over-worked through the absence/rarity of epigeic and anecic earthworms that have well known sensitivity to tillage. Reducing the sampling intensity to five soil pits (e.g. #30minworms) and changing the sub-optimal threshold to <20%, shows good agreement to the 10-pit survey ≤10% category threshold (S5 Table). An alternative metric is to rate the soil health of a field based on earthworm numbers at a sampling intensity of one soil pit per field as proposed for the AHDB soil scorecard[31]. This survey indicates that between 68–88% fields could be categorized as ‘depleted’ through to ‘active’ (S6 Table). In comparison a sampling intensity of five soil pits per field provided average earthworm count data that was in good agreement with these data calculated at 10 soil pits per field (S6 Table), and 20% of fields would be categorized as ‘depleted’ at this sampling intensity.

**Fig 4:**
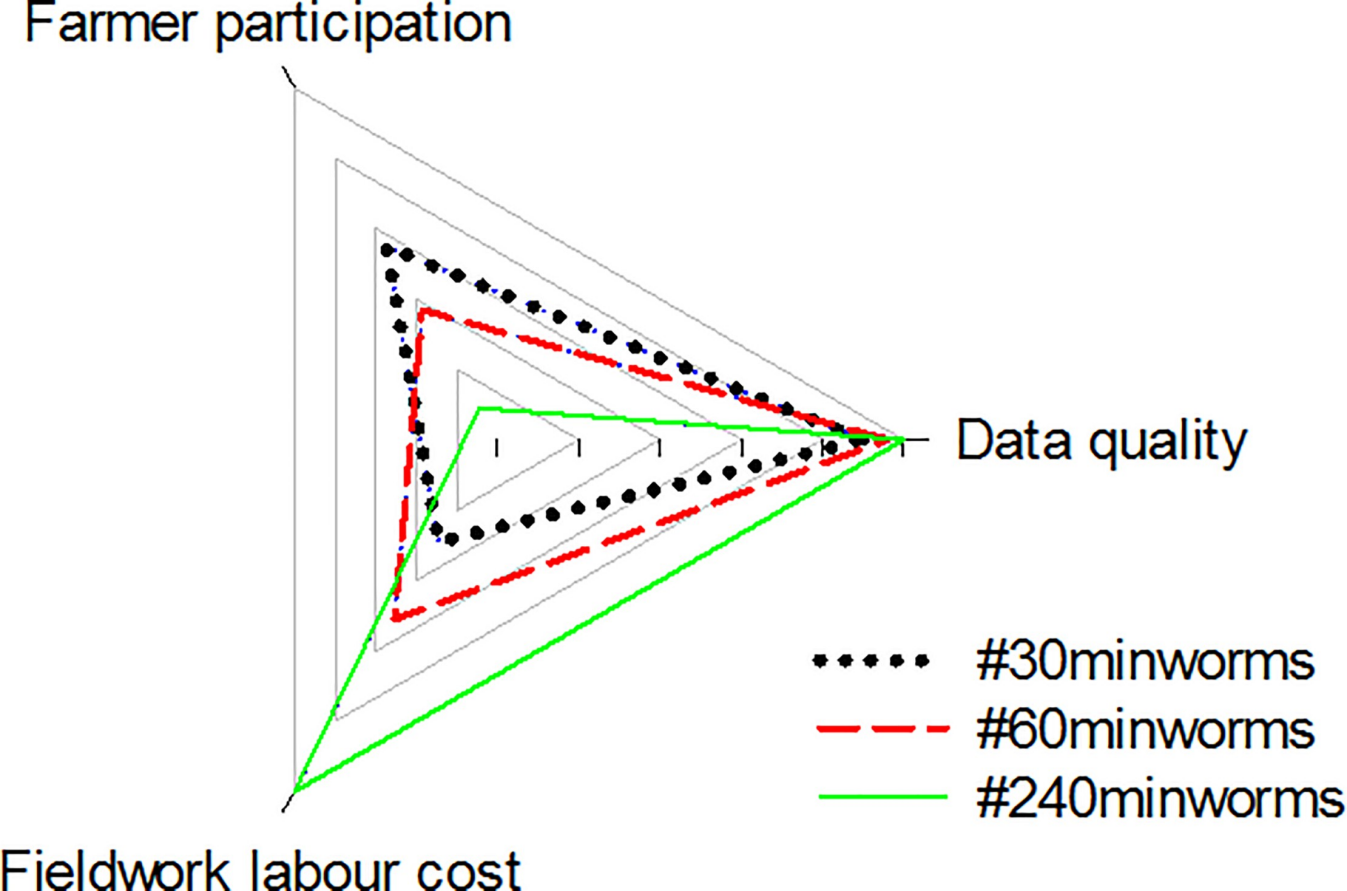
Trade-offs between earthworm fieldwork effort (30–240 mins) and data quality, farmer participation levels and labour costs.

The trade-off was estimated using data quality (% false negatives), participation (scaling booklet requests to 100% and actual survey time to 100%) and cost (using an intensive 20 pits x 10 minutes earthworm fieldwork set at 100%), indicates that a five-pit field survey has significant potential (Fig 4). An average #30minworms field survey (10.9 ± 0.8 ha^-1^) would incur £16–48 in fieldwork costs depending on labour type (farmer or outsourced). Scaling to #30minworms of the whole arable area (52%) of an average farm in England (85 ha) would range between £65–196 in fieldwork costs depending on labour type. Significant regional variations in farm costs would be expected; fieldwork costs on the arable area on an average farm in the North East being £23–70, where the East of England would cost £134–401; reflecting farm size and arable cropping area. Nationally, a #30minworms survey of the entire 4.74 million hectares of land under arable cropping would have fieldwork costs at £7 million (farmer participation) to £21 million (outsourced) per survey.

## Discussion

The pilot #60minworms study effectively mobilised farmers to reach the target of 100 fields (Fig 1). It was hypothesised that the workshops and newsletters would lead to the highest recruitment and participation rates due to a direct interaction and targeted approach (requiring a high time and cost), but posed a risk of location bias i.e. small geographic area monitoring. However, these channels had no impact on participation. Twitter, Farmers Weekly and The Farmers Forum were the most effective channels for recruitment. Twitter and Farmers Weekly recruits had exceptional participation and engagement rates, demonstrating the potential importance of these media channels to achieving soil security in agriculture. The impact of e.g. Twitter and Farmers Weekly over that of the isolated workshops and newsletters; with a further benefit of the wide geographic survey spread (Fig 3) could be explained by the high interest in community science that was identified at the #60minworms workshop (post sampling), with participants placing high value on others’ results, data collection abilities and motivations for sampling (Fig 2). The community concept is further corroborated by the primary application of monitoring being to compare results within and between farms (64%), and a high (87%) interest in annual earthworm results from scientific national capability field trials e.g. Broadbalk indicating the potential to amplify both spatial and temporal soil health monitoring over and above what is achievable by these groups individually. Future developments that prioritize quick assessment protocols to enhance participation rates (farmers and number of fields), such as a #30minworms survey (Fig 4) would likely be the most useful to farmers, as most participants (57%) would change their soil management practices as a result earthworm monitoring results. This is in agreement with the ‘monitoring effect’, which is a confounding factor for gauging biodiversity[5], but is aligned with the DEFRA aspiration of sustainable soils by 2030. The absence of interest in regional data agrees with the primary interest in soil management (Fig 2), and may explain the low participation rates by farmers in ecological earthworm surveys to date. At a national scale, £14 million pounds per #30minworms survey could be saved by mobilising farmers; demonstrating the potential high value of farmer input to achieving sustainable farmland soil policy. Developing a robust method is the first step to farmland soil monitoring, and may enable earthworms to be used as a biological indicator by DEFRA to achieve policy aspirations of sustainable soils by 2030

The #60minworms method is a protocol validated for farmer applications, with feedback indicating high levels of trust, value and satisfaction by the participants (Figs 2 and 3). There were no indications of significant sampling bias or problems in method compliance, however a key training need in earthworm identification skills was identified e.g. 46% participants were not confident in their earthworm adult/juvenile separation and identification skills, but a significant interest in gaining this skill (Fig 1). Farmer feedback led to modifications and improvement to the methodology and results presentation (S1 Protocol).

The findings from the #60minworm survey showed that earthworms are ubiquitous in UK farmland, with 100% presence recorded on the majority (77%) fields. The majority of these fields are managed under conventional agriculture (i.e. pesticides and inorganic fertilisers are used), and intensive cultivations have dominated crop establishment practices in England[15]. There was a significant (*p<* 0.05) impact of tillage on all parameters except endogeic earthworm presence (S1 Fig, S4 Table). The survey revealed that there were no sightings of epigeic and anecic earthworm species, which are the two most sensitive ecological groups to tillage[13], on 21% and 16% fields respectively, and they were rare (≤10% presence) on a further 8% and 11% fields (S5 Table). This is a cause for concern given the slow earthworm population recovery rates under changed management practices [32], and slow anecic earthworm reproduction rates, for example 8 cocoons per earthworm per year, with a 60 week development time [33]. No earthworm hotspots were detected in almost half (46%) fields, where ≥ 16 worms per pit are linked to significant benefits in plant productivity (although this is highly dependent on a number of factors so does not have a strong interpretative value)[6]. At these measured on-farm population levels, these data indicate the majority of UK farmland soils have satisfactory earthworm presence and abundance, but there is potential to increase the presence of these ecosystem engineers to better support both food security, but also wider earthworm-mediated ecosystem services such as native wildlife prey, soil aggregation and water infiltration; associated with soil security.

The ‘traffic light’ for results interpretation here was ranked as useful (36%), but has an escalating error in categorizing earthworms at ≤10 sample pits, which could hinder participation whilst increase costs of monitoring (Fig 4). Simplification is needed for a #30minworms survey, for example simply a ‘sub-optimal’ or ‘satisfactory’ score, the former indicated by < 20% (b) epigeic, (c) endogeic and (d) anecic earthworm (or midden/vertical burrow) presence), would mitigate the problem of ‘false-negatives’ as both absent and rare (≤10% presence) are within this ‘sub-optimal’ category (S5 Table). To aid the identification of exceptional earthworm populations for case-studies of soil management practices; Gold (100%), Silver (≥80%) and Bronze (≥60%) ecological group presence could be used; of which 15% of fields in this survey would have achieved a Gold or Silver rating. The value of ‘earthworm numbers’ is unclear, for example, earthworm numbers are linked to benefits in plant productivity, but this impact depends on soil texture, crop type and fertilisation regime [6], confounding the interpretative power of this parameter. In terms of quality control of this measurement, there is a high labour cost (doubling of the hand-sorting assessment to 10-minutes for accuracy to improve the detection of juvenile worms), although a correction factor of 1.6 worms pit^-1^ could be used; and given the high variability (up to 28-fold) between soil pits, multiple soil pits are needed to provide a robust earthworm number estimate for specific moment in time (S6 Table) and this is a parameter with high annual variability (S1 Table).

General strategies to increase the presence of earthworms would be to reduce tillage frequency and intensity (S1 Fig), however the impact of soil management activities is subject to local conditions (S3 Table), and monitoring is an essential component to realising soil health in practice. One strategy that provided mixed impact on earthworm populations is organic matter management (S2 Fig, S4 Table). Three types of organic matter management were recorded, with straw retention or manuring having no significant (*p >* 0.05) impact on the presence of the ecological groups. However, cover cropping significantly (*p <* 0.05) increased the presence of anecic earthworms only (S2 Fig, S4 Table). Thus, there was little evidence for organic matter management mitigating tillage impacts on earthworm populations. Identifying ‘at risk’ fields (up to 42% fields in this survey), through the absence/rarity of epigeic and anecic earthworms, provides, for the first time, the opportunity for management intervention strategies to mitigate the effects of over-worked soils and support the DEFRA policy aspiration of sustainable soils by 2030.

## Supporting information

S1 Table Community structure of earthworms from an annual assessment. Survey analysis using the hand-sorting data from multiple annual assessments on a field trial managed under different organic matter rates and types. Despite large fluctuations in earthworm numbers, there was a consistent community structure.
(PDF)

S2 Table Community structure of earthworms from a seasonal assessment. Limited seasonal variation in earthworm community structures was detected on the AHDB Strategic Farm East in Autumn 2017 and Spring 2018 (n = 20 pits per field)
(PDF)

S3 Table Field characteristics from top and bottom on the survey. Field characteristics of the top and bottom 10 fields in the #60minworms survey.
(PDF)

S4 Table Statistical data analysis of tillage. P values from one-way ANOVA analyses of the #60minworms data set showing the significance of tillage on all parameters except endogeic presence. In comparison organic matter management practices of straw retention, cover cropping or manuring had little significant impact on earthworm parameters, with only cover cropping having a significant impact on anecic earthworm presence.
(PDF)

S5 Table Summary of earthworm communities. (a) The percentage of fields under earthworm ecological group presence categories, where no sightings are 0% and may indicate a local extinction; and a likely presence is > 66%, indicating there is good evidence for their presence based on 10 soil pits. (b) Fields with a sub-optimal ≤10% presence (absent, rare) presence of earthworm ecological groups. (c) The percentage of fields under earthworm ecological group presence categories, where no sightings are 0% and may indicate a local extinction; and a likely presence is > 66%, indicating there is good evidence for their presence based on 5 soil pits.
(PDF)

S6 Table Comparison of 5 soil pits compared to 10 soil pits. The field interpretation of earthworm counts at five pits compared to 10 pits is similar. However, there is high uncertainty at a low sampling intensity (one sample pit per field) as most fields (68–86%) contain at least one pit (out of 10 pits) at each of the earthworm categories. This indicates that there is a considerable risk in over-estimating sub-optimal earthworm populations.
(PDF)

S1 Fig Tillage and earthworm community. The #60minworm survey results showed a negative impact (p < 0.05*) of tillage on earthworm presence (a, b, d, e) and numbers (f) (except endogeic presence).
(PDF)

S2 Fig Organic matter management and earthworm community. The #60minworm survey found no significant (*p >* 0.05) impacts from straw retention or manuring management practices. Cover cropping had no significant (*p >* 0.05) impact on epigeic or endogeic earthworm presence, but a beneficial impact (*p <* 0.05*) on anecic earthworm presence.
(PDF)

S1 Protocol Survey sheets used by participants. #60minworms Pilot study booklet and the new #30minworms booklet.
(PDF)

S1 Raw data Spreadsheet of earthworm data. Excel spreadsheet of earthworm data from each field.
(XLSX)
