# Supplementary material for: Soil health pilot study in England: Outcomes from an on-farm earthworm survey
Source: PLoS One. 2019 Feb 20;14(2):e0203909. doi: 10.1371/journal.pone.0203909 (PMC6382109; doi:10.1371/journal.pone.0203909)
Supplement: S1 Table — Survey analysis using the hand-sorting data from multiple annual assessments on a field trial managed under different organic matter rates and types. Despite large fluctuations in earthworm numbers, there was a consistent community structure. (PDF) [file pone.0203909.s001.pdf]

Table S1: Survey analysis using the hand-sorting data from multiple annual assessments on field trials managed under different organic matter rates and types. Despite large fluctuations in earthworm numbers, there was a consistent community structure.

Field number	Soil pits	>1 earthworm per pit (%)	**Presence epigeic (%)**	**Presence endogeic (%)**	**Presence anecic (%)**	>400 worms m^2 per pit (%)	Earthworms (millions ha^{-1})
1 (2013, before)	10	100	0	80	0	10	1.6
Split field experiment design							
1 (2014, Spring Barley)	24	100	0	96	0	58	5.7
1 (2016, Winter Wheat)	96	100	0	77	7	4	1.9
Split field experiment design							
1 (2014, Winter Oil Seed Rape)	24	100	0	83	0	79	6.6
1 (2016, Spring Barley)	96	97	0	58	2	0	1.4
Unsplit field experiment design							
1 (2018, Winter Oil Seed Rape)	40	100	0	82	7	10	2.0
