# Supplementary material for: Soil health pilot study in England: Outcomes from an on-farm earthworm survey
Source: PLoS One. 2019 Feb 20;14(2):e0203909. doi: 10.1371/journal.pone.0203909 (PMC6382109; doi:10.1371/journal.pone.0203909)
Supplement: S2 Table — Limited seasonal variation in earthworm community structures was detected on the AHDB Strategic Farm East in Autumn 2017 and Spring 2018 (n = 20 pits per field) (PDF) [file pone.0203909.s002.pdf]

Table S2: Limited seasonal variation in earthworm community structures was detected on the AHDB Strategic Farm East in Autumn 2017 and Spring 2018 (n = 20 pits per field)

	**Epigeic presence**		**Endogeic presence**		**Anecic presence**	
	Autumn 17	*Spring 18*	*Autumn 17*	*Spring 18*	*Autumn 17*	*Spring 18*
**Kells**	10%	20%	35%	65%	10%	20%
**West Farm**	5%	5%	70%	40%	35%	20%
**Wally's**	15%	35%	60%	35%	20%	20%
**Big Lawn**	10%	30%	100%	40%	30%	20%
**Barn Field**	5%	0%	85%	70%	30%	30%
**Retters**	5%	0%	90%	30%	25%	25%
**Rushbottom**	30%	25%	95%	90%	35%	40%
**Apple Tree**	10%	10%	90%	90%	40%	40%
**Shrubbery**	15%	15%	75%	90%	70%	70%
