# Supplementary material for: Soil health pilot study in England: Outcomes from an on-farm earthworm survey
Source: PLoS One. 2019 Feb 20;14(2):e0203909. doi: 10.1371/journal.pone.0203909 (PMC6382109; doi:10.1371/journal.pone.0203909)
Supplement: S3 Table — Field characteristics of the top and bottom 10 fields in the #60minworms survey. (PDF) [file pone.0203909.s003.pdf]

Table S3: Field characteristics of the top and bottom 10 fields in the #60minworms survey.

	**Top 10 fields**	**Lowest 10 fields**
**Soil textures**	Clay, silt, sand	Clay, silt, sand, chalk
**Tillage**	NoTill, MinTill, Plough	MinTill, Plough
**Straw retention (%)**	90	20
**Cover crop (%)**	40	40
**Manured (or other) (%)**	30	50
**Winter cereals (%)**	60	50
