# Supplementary material for: Soil health pilot study in England: Outcomes from an on-farm earthworm survey
Source: PLoS One. 2019 Feb 20;14(2):e0203909. doi: 10.1371/journal.pone.0203909 (PMC6382109; doi:10.1371/journal.pone.0203909)
Supplement: S4 Table — P values from one-way ANOVA analyses of the #60minworms data set showing the significance of tillage on all parameters except endogeic presence. In comparison organic matter management practices of straw retention, cover cropping or manuring had little significant impact on earthworm parameters, with only cover cropping having a significant impact on anecic earthworm presence. (PDF) [file pone.0203909.s004.pdf]

Table S4: P values from one-way ANOVA analyses of the #60minworms data set showing the significance of tillage on all parameters except endogeic presence. In comparison organic matter management practices of straw retention, cover cropping or manuring had little significant impact on earthworm parameters, with only cover cropping having a significant impact on anecic earthworm presence.

**Parameter**	**Tillage**	**Straw retained**	**Cover crop**	**Manured**
Number of fields	*126*	*Yes: 49, No: 66*	*Yes: 28, No: 87*	*Yes:37, No :78*
**Widespread presence**	**0.006**	0.060	0.218	0.219
**Epigeic presence**	**0.010**	0.286	0.730	0.537
**Endogeic presence**	0.123	0.674	0.622	0.583
**Anecic presence**	**0.001**	0.217	**0.027**	0.178
**Hotspot presence**	**0.001**	0.540	0.209	0.143
**Number of earthworms**	**0.001**	**0.004**	0.182	0.416
