# Supplementary material for: Soil health pilot study in England: Outcomes from an on-farm earthworm survey
Source: PLoS One. 2019 Feb 20;14(2):e0203909. doi: 10.1371/journal.pone.0203909 (PMC6382109; doi:10.1371/journal.pone.0203909)
Supplement: S5 Table — (a) The percentage of fields under earthworm ecological group presence categories, where no sightings are 0% and may indicate a local extinction; and a likely presence is > 66%, indicating there is good evidence for their presence based on 10 soil pits. (b) Fields with a sub-optimal ≤10% presence (absent, rare) presence of earthworm ecological groups. (c) The percentage of fields under earthworm ecological group presence categories, where no sightings are 0% and may indicate a local extinction; and a likely presence is > 66%, indicating there is good evidence for their presence based on 5 soil pits. (PDF) [file pone.0203909.s005.pdf]

Table S5a: The percentage of fields under earthworm ecological group presence categories, where no sightings are 0 % and may indicate a local extinction; and a likely presence is > 66 %, indicating there is good evidence for their presence based on 10 soil pits.

Ten soil pits	**Field survey presence of earthworm ecological groups**			
	0 %	≤20 %	>20 – 66 %	>66%
Epigeic adult earthworms	21 %	16 %	29 %	35 %
Endogeic adult earthworms	2 %	15 %	30 %	53 %
Anecic adult earthworms	14 %	23 %	36 %	27 %

Table S5b: Fields with a sub-optimal ≤10 % presence (absent, rare) presence of earthworm ecological groups

Ten soil pits	
	**≤10 %**
Epigeic adult earthworms	**29 %**
Endogeic adult earthworms	**11 %**
Anecic adult earthworms	**25 %**

Table S5c: The percentage of fields under earthworm ecological group presence categories, where no sightings are 0 % and may indicate a local extinction; and a likely presence is > 66 %, indicating there is good evidence for their presence based on 5 soil pits.

Five soil pits	**Field survey presence of earthworms**			
	**0 %**	20%	>20 – 66 %	>66%
Epigeic adult earthworms	**30 %**	13 %	23 %	33 %
Endogeic adult earthworms	**10 %**	16 %	23 %	52 %
Anecic adult earthworms	**23 %**	27 %	30 %	30 %
