# Supplementary material for: Soil health pilot study in England: Outcomes from an on-farm earthworm survey
Source: PLoS One. 2019 Feb 20;14(2):e0203909. doi: 10.1371/journal.pone.0203909 (PMC6382109; doi:10.1371/journal.pone.0203909)
Supplement: S6 Table — The field interpretation of earthworm counts at five pits compared to 10 pits is similar. However, there is high uncertainty at a low sampling intensity (one sample pit per field) as most fields (68–86%) contain at least one pit (out of 10 pits) at each of the earthworm categories. This indicates that there is a considerable risk in over-estimating sub-optimal earthworm populations. (PDF) [file pone.0203909.s006.pdf]

Table S6: The field interpretation of earthworm counts at five pits compared to 10 pits is similar. However, there is high uncertainty at a low sampling intensity (one sample pit per field) as most fields (68 – 86 %) contain at least one pit (out of 10 pits) at each of the earthworm categories. This indicates that there is a considerable risk in over-estimating sub-optimal earthworm populations.

	**Field soil health category**		
	1 pit	5 pits (mean)	10 pits (mean)
Active<br>(>8 worms per pit)	78 % fields	55 % fields	53 % fields
Intermediate<br>(4 – 8 worms per pit)	68 % fields	25 % fields	27 % fields
Depleted<br>(<4 worms per pit)	86 % fields	20 % fields	18 % fields
