# Supplementary material for: Soil health pilot study in England: Outcomes from an on-farm earthworm survey
Source: PLoS One. 2019 Feb 20;14(2):e0203909. doi: 10.1371/journal.pone.0203909 (PMC6382109; doi:10.1371/journal.pone.0203909)

Figure S1: The #60minworm survey results showed a negative impact ($p < 0.05$*) of tillage on earthworm presence (a, b, d, e) and numbers (f) (except endogeic presence).

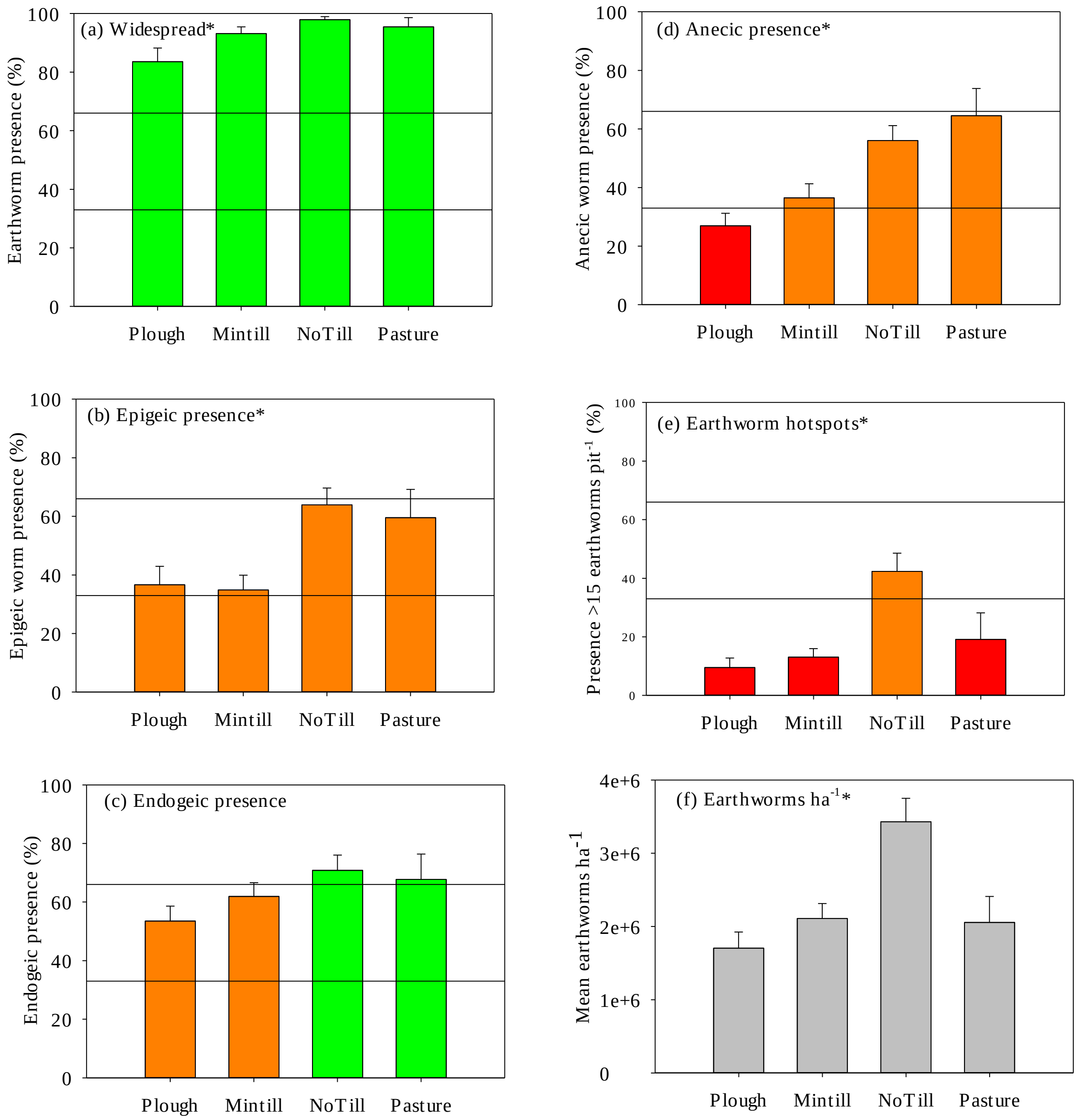

Supplement: S1 Fig — The #60minworm survey results showed a negative impact (p < 0.05*) of tillage on earthworm presence (a, b, d, e) and numbers (f) (except endogeic presence). (PDF) [file pone.0203909.s007.pdf]
