# Supplementary material for: Soil health pilot study in England: Outcomes from an on-farm earthworm survey
Source: PLoS One. 2019 Feb 20;14(2):e0203909. doi: 10.1371/journal.pone.0203909 (PMC6382109; doi:10.1371/journal.pone.0203909)

Figure S2: The #60minworm survey found no significant (p > 0.05) impacts from straw retention or manuring management practices. Cover cropping had no significant (p > 0.05) impact on epigeic or endogeic earthworm presence, but a beneficial impact (p < 0.05*) on anecic earthworm presence.

Epigeic earthworms:

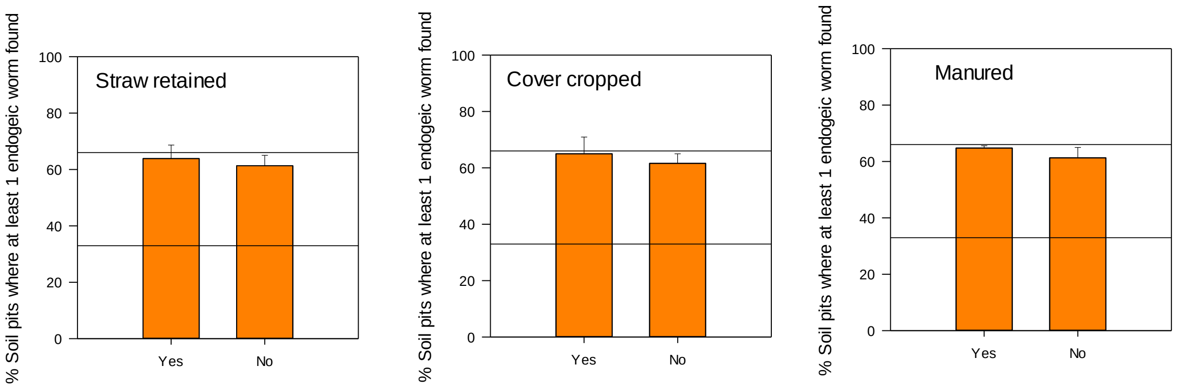

Endogeic earthworms:

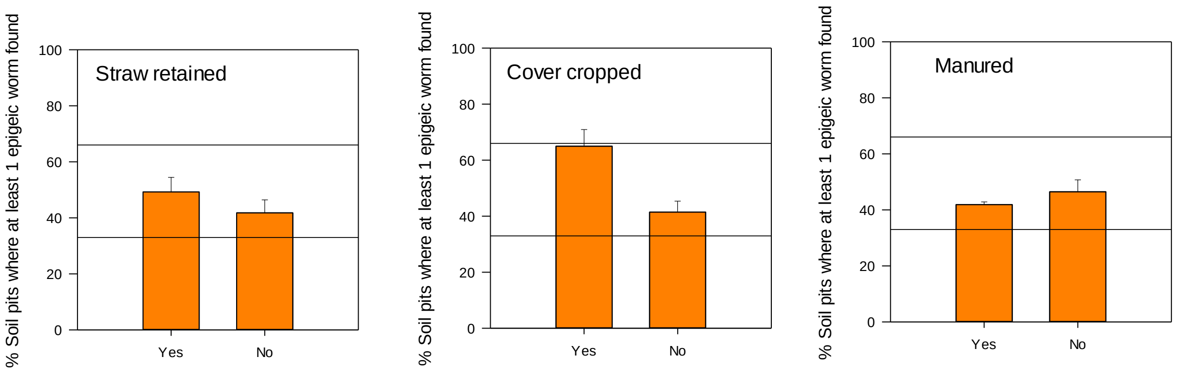

Anecic earthworms

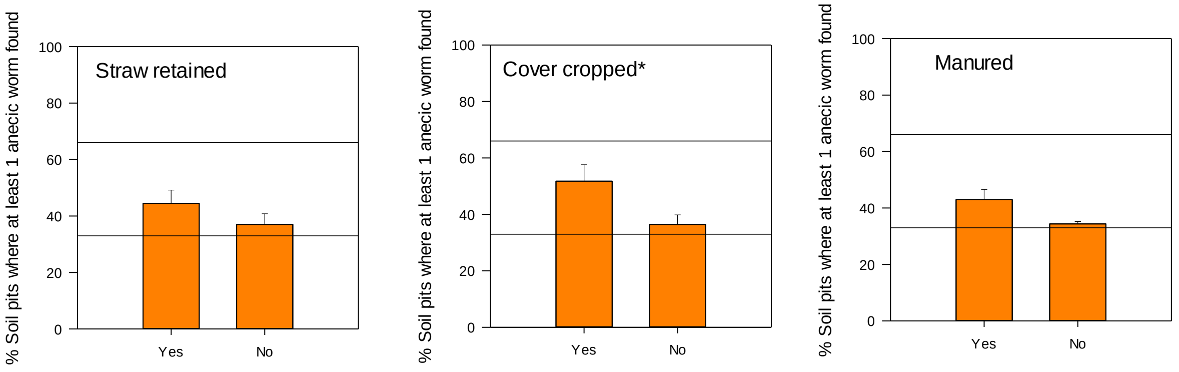

Supplement: S2 Fig — The #60minworm survey found no significant (p > 0.05) impacts from straw retention or manuring management practices. Cover cropping had no significant (p > 0.05) impact on epigeic or endogeic earthworm presence, but a beneficial impact (p < 0.05*) on anecic earthworm presence. (PDF) [file pone.0203909.s008.pdf]
