# Supplementary material for: Soil health pilot study in England: Outcomes from an on-farm earthworm survey
Source: PLoS One. 2019 Feb 20;14(2):e0203909. doi: 10.1371/journal.pone.0203909 (PMC6382109; doi:10.1371/journal.pone.0203909)
Supplement: S1 Protocol — #60minworms Pilot study booklet and the new #30minworms booklet. (PDF) [file pone.0203909.s009.pdf]

60 min worms

Earthworm image here

Good for worms, good for soils, good for you

Booklet number

Soil testing 11/03/18 to 30/04/18

@wormscience

Method 02/18

Safety information: This is an outdoor activity, digging holes can be strenuous, cover open wounds before handling soils, and wash your hands after the assessment.

Equipment

Spade & ruler | *Mat* | *Pot for worms* | *Bottle water* | *Booklet & pen*

Procedure

10 soil pits per field using standard W shape field sampling

1) Dig out a 20 cm x 20 cm x 20 cm soil pit and place soil on mat (30 sec)

2) Hand-sort soil (5-minutes, unless compacted or clayey which takes longer), placing each whole earthworm into the pot

3) Count the total numbers of earthworms and write down

4) Separate earthworms into adults (only a few) and return juveniles to soil pit. May need to rinse worms with water to detect if a saddle is present

Adult (has saddle)
Saddle is the reproductive ring
Thickened area near head
Often different colour to body

Juvenile (no saddle)

Adult

5) Count the numbers of each type of adult earthworm (key shown) and write down

6) Return worms to soil pit and back fill with soil

7) Repeat steps 1 - 6, until 10 soil pits per field have been assessed

8) Please send me your data by post or email, and I will calculate and return results.
Postal address: Dr J. Stroud, Rothamsted Research, Harpenden, Herts. AL5 2JQ

Type 1: Epigeic

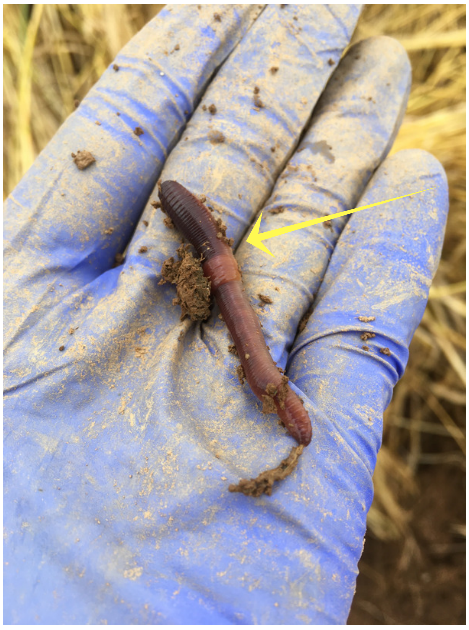

Small (matchstick) size
Red bodied worm
Often fast moving

Type 2: Endogeic

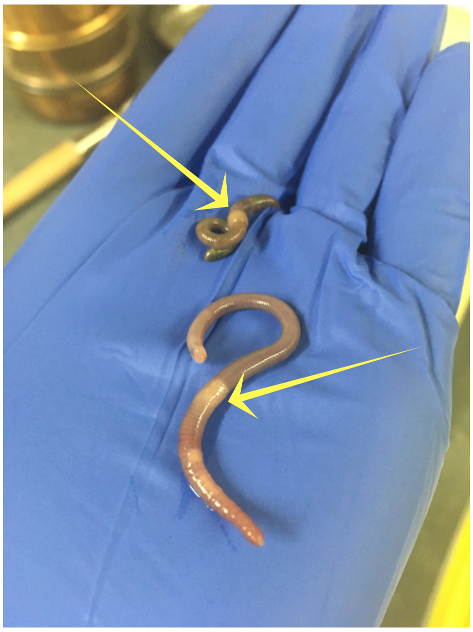

Small - Medium size
Not red
Pale or green colours

Type 3: Anecic

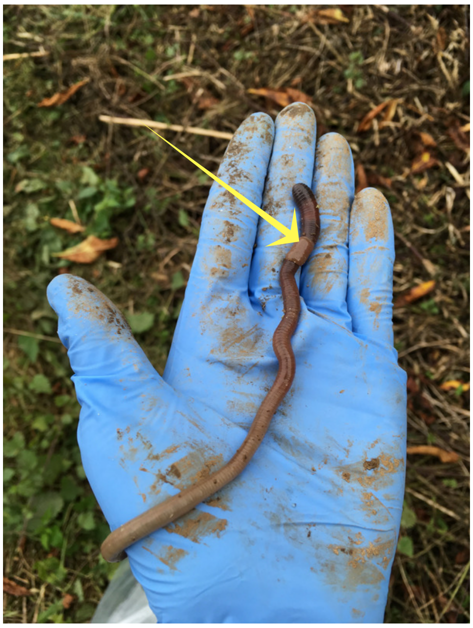

Large (pencil) size
Red head & fat body or
black head & long body

Data table for booklet number ....

Field name: .... Field Size: .... Current crop: ....

Was straw retained? YES/NO Tillage ? PLOUGH/MINTILL/NOTILL

Cover crop prior? YES/NO Manure/compost etc. this year?......................

	Pit 1	Pit 2	Pit 3	Pit 4	Pit 5	Pit 6	Pit 7	Pit 8	Pit 9	Pit 10
Total number of worms										
Number of type 1: Epigeic worms										
Number of type 2: Endogeic worms										
Number of type 3: Anecic worms										

Photo page & send to: jacqueline.stroud@rothamsted.ac.uk

Traffic-light based tracker to signal healthy soil management practices

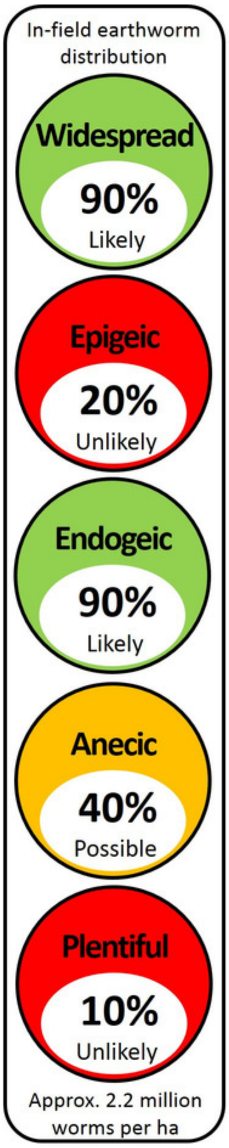

Widespread

- Indicates the likelihood of a ubiquitous earthworm population in-field
- Sensitive to soil physical (e.g. waterlogging) and chemical (e.g. pH) properties
- Good distributions are important for field-wide worm functions

Epigeic

- Indicates the likelihood of finding epigeic earthworms in-field
- Epigeic populations are sensitive to tillage and organic matter management
- Important role in carbon cycling and prey for native birds e.g. song thrush

Endogeic

- Indicates the likelihood of finding endogeic earthworms in-field
- Endogeic populations are sensitive to organic matter management
- Important roles in soil aggregation and nutrient mobilisation for plants

Anecic

- Indicates the likelihood of finding anecic earthworms in-field
- Anecic populations are sensitive to tillage and organic matter management
- Important role in forming deep burrows for plant roots and water infiltration

Plentiful

- Indicates the likelihood of finding high numbers of earthworms in-field
- Worm numbers fluctuate seasonally (weather) and annually (crop rotation)
- Worm populations above 400 per m^2 significantly benefit plant productivity

Approximate number of soil ecosystem engineers per hectare

If you can measure it, you can manage it

I believe that our soils could be better: better at supporting crop production; better at supporting native fauna; and better at supporting carbon storage. To best unlock the potential of soils I think better measurements are needed, which is why I designed this test. Decisions that are made above the ground influence the millions of earthworms that are engineering the soil ecosystem below the ground. Spending their entire lives eating, burrowing and breeding in soils, with sensitivities to pH, waterlogging, compaction, rotations, tillage and organic matter management – it's easy to understand why earthworms are a candidate national soil health indicator. Practically, the key is knowing what you have, in order to make it the best that it can be. Thank-you for getting involved in soil science! Jackie.

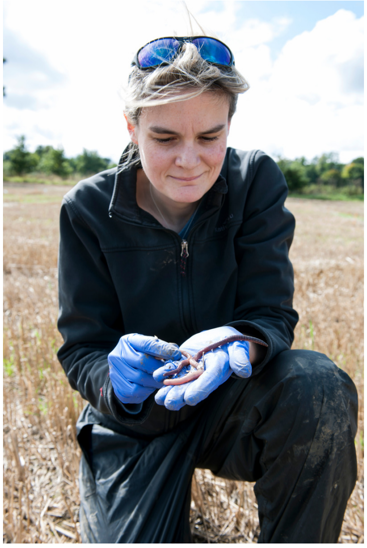

#60minworms

Acknowledgements: I'd like to thank Jeremy LeLean (SSP) for his support to create this leaflet, and the AHDB KE team for our discussions, on-farm testing of method v.10/17 and feedback.

#30minworms

On-farm soil health assessment booklet

Best sampling time: Autumn - Spring

Earthworm images here

www.wormscience.org

@wormscience

#30minworms method

Safety information: This is an outdoor activity, digging holes can be strenuous, cover open wounds before handling soils, and wash your hands after the assessment.

Equipment

Spade & ruler | *Mat* | *Pot for worms* | *Bottle water* | *Booklet & pen*

Procedure

5 soil pits per field using standard W shape field sampling

1) Dig out a 20 cm x 20 cm x 20 cm soil pit and place soil on mat (30 sec)

2) Hand-sort soil (5-minutes), placing each whole earthworm into the pot. Note if pencil size vertical burrows are present and tick/cross on the results sheet

3) Count the total number (adults and juveniles) of earthworms and write down

4) Separate earthworms into adults (only a few) and return juveniles to soil pit. May need to rinse worms with water to detect if a saddle is present

Adult (has saddle)
Saddle is the reproductive ring
Thickened area near head
Often different colour to body

Juvenile (no saddle)

Adult

5) Count the numbers of each type of adult earthworm (key shown) and write down.

6) Return worms to soil pit and back fill with soil

7) Check the soil surface for the presence of middens (key shown)

8) Repeat steps 1 - 7, until 5 soil pits per field have been assessed

9) Please input your data at www.wormscience.org for results analysis

ID Step 1 of 2: Separate Adult vs Juveniles

Only adults have a saddle or belt (worm at top of each picture)

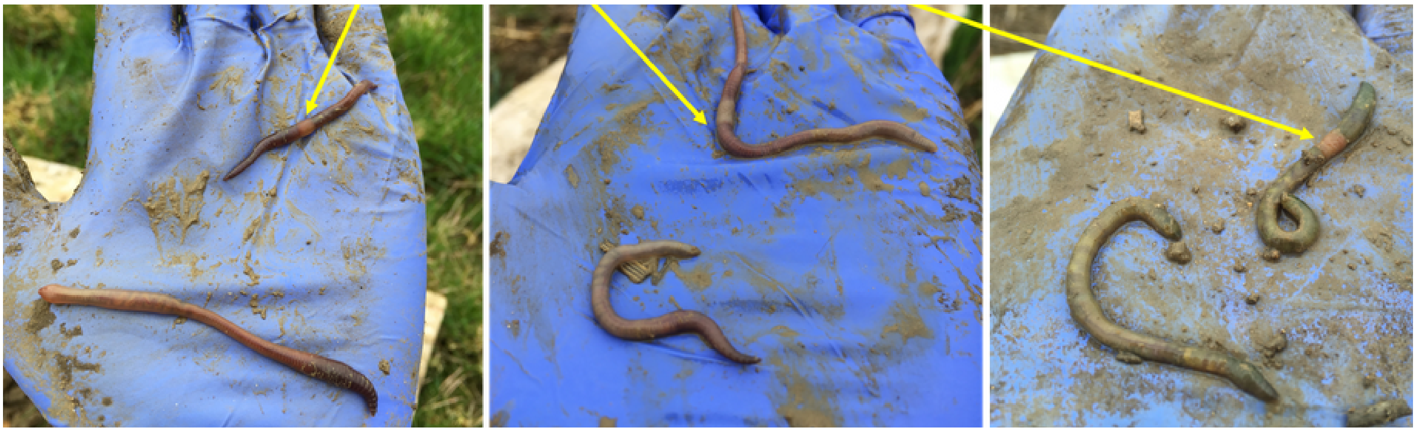

ID Step 2 of 2: Identify adult ecological group

Practice your ID skills on the worm ID quiz at wormscience.org

Type 1: Surface worms e.g. *Lumbricus castaneus, Lumbricus rubellus*

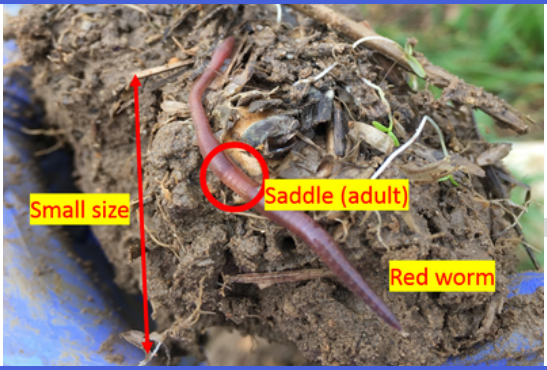

Small (matchstick) size

<8 cm when not moving

Red bodied worm

What do they do? : Breakdown surface litter and good food source for native birds

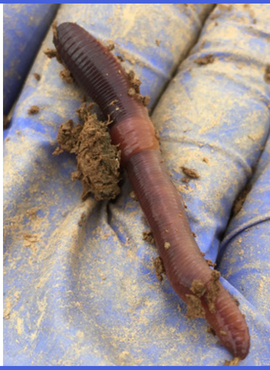

Type 2: Topsoil worms e.g. *Aporrectodea caliginosa, Allolobophora chlorotica*

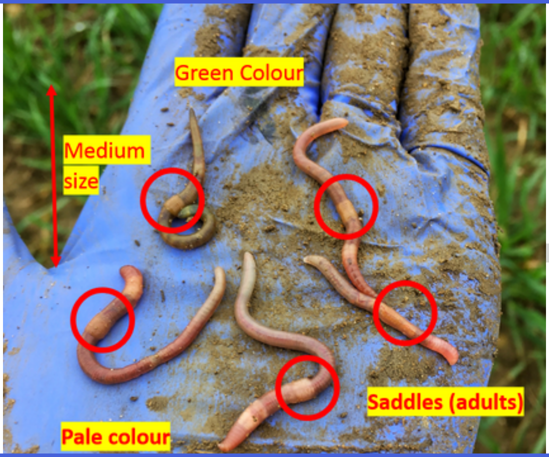

Small - Medium size

Pale worms: grey, pink or dark green colour

What do they do? Mix soil & mobilise nutrients for plant uptake, supporting crop productivity

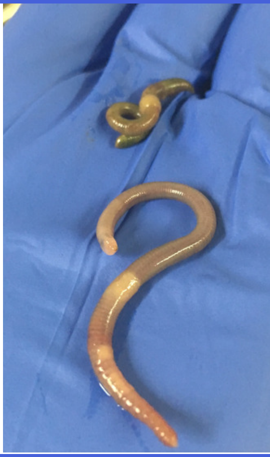

Type 3: Deep burrowers e.g. *Lumbricus terrestris, Aporrectodea longa*

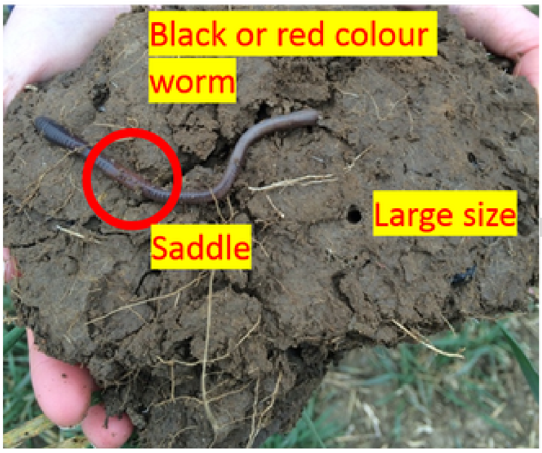

Large (pencil) size

Heavily pigmented (red or black headed earthworms)

What do they do? These are the 'drainage' worms - can form 2 m vertical burrows, helping with water infiltration and deep plant rooting

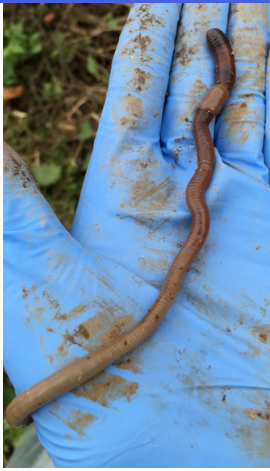

Deep burrowing earthworm presence

Deep burrowers may not be captured in the topsoil so look out for these indicators of their presence and note down on the data table

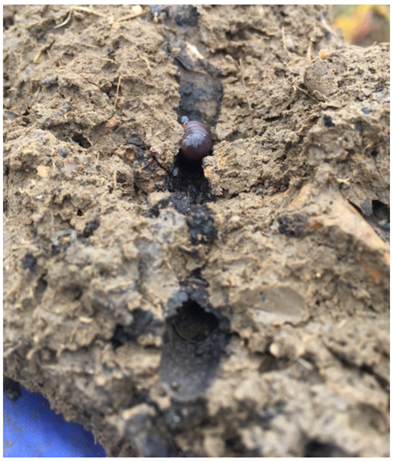

Pencil size vertical burrow

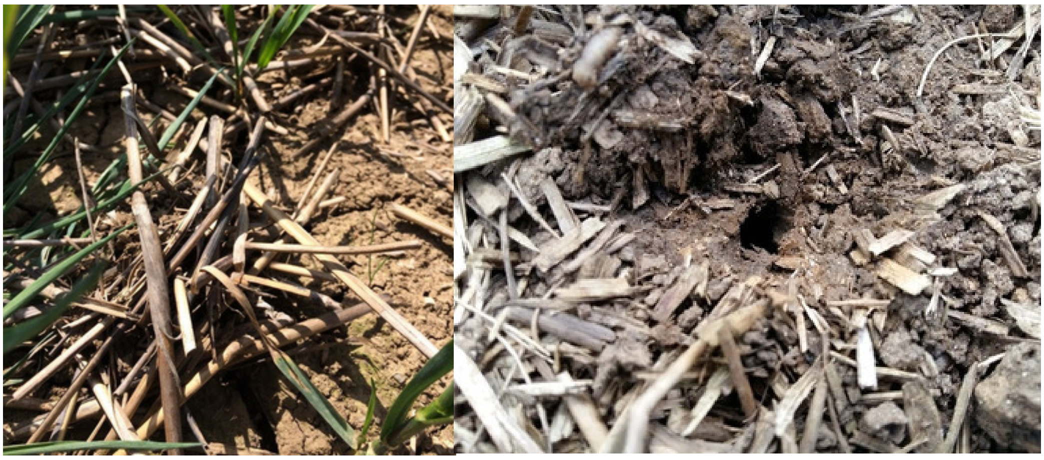

Midden made by a deep burrowing earthworm. This is a pile of straw or stones overlying a permanent burrow.

#30minworms data table:

Field name: .... Field Size (ha): .... Crop: .... Date:..................

Was straw retained? YES/NO Tillage ? PLOUGH/MINTILL/NOTILL

Cover crop prior? YES/NO Manure/compost etc. this year?.....................

	Pit 1	Pit 2	Pit 3	Pit 4	Pit 5
Large vertical burrows or middens present?					
Total number of juvenile AND adult worms					
Total number of adult surface worms					
Total number of adult topsoil worms					
Total number of adult deep burrowers					

Please enter your data at www.wormscience.org for results analysis
